# Social Media Use and Depression in Adolescents: A Scoping Review

**DOI:** 10.3390/bs13060475

**Published:** 2023-06-06

**Authors:** Layan Azem, Rafaa Al Alwani, Augusto Lucas, Balqes Alsaadi, Gilbert Njihia, Bushra Bibi, Mahmood Alzubaidi, Mowafa Househ

**Affiliations:** College of Science and Engineering, Hamad Bin Khalifa University, Doha 34110, Qatarmhouseh@hbku.edu.qa (M.H.)

**Keywords:** social media, social network, depression, adolescents, SNS

## Abstract

This scoping review aimed to investigate the association between depression and social media use among adolescents. The study analyzed 43 papers using five databases to identify articles published from 2012 to August 2022. The results revealed a connection between social media use and depression, as well as other negative outcomes such as anxiety, poor sleep, low self-esteem, and social and appearance anxiety. Surveys were the most used study strategy, with multiple common scales applied to assess depression, social media use, and other factors such as self-esteem and sleep quality. Among the studies, eight reported that females who use social media showed higher depression symptoms than males. This scoping review provides an overview of the current literature on the relationship between social media use and depression among adolescents. The findings emphasize the importance of monitoring social media use and providing support for individuals struggling with depression. However, more research is needed to better understand the factors contributing to this relationship and to develop more standardized assessment methods.

## 1. Introduction

### 1.1. Background

The term “social media” refers to websites and applications that emphasize communication, community-based input, interaction, content sharing, and collaboration [1]. There has been an increase in depressed adolescents in the US since 2012 [2]. Simultaneously, social media became more engaging which led to an increase in social media users [2]. According to the American Psychiatric Association [3], depression is a serious medical condition that can have a negative impact on how you feel, think, and act. Sadness and/or a loss of interest in previously enjoyable activities are symptoms of depression [3]. It can hinder your ability to function at work and home and cause various emotional and physical issues [3]. The World Health Organization (WHO) has estimated that depression affects 3.8% of the global population which corresponds to 280 million people [4]. The WHO defines adolescents as people between the ages of 10 and 19 [5]. Adolescence is the stage of life between childhood and adulthood. It is a distinct period in human development and crucial for setting the groundwork for long-term health. Teenagers grow quickly regarding their physical, cognitive, and emotional development. This impacts their emotions, thoughts, decisions, and interactions with others and their environment [5]. There has been an increase in the number of depressed adolescents over the past decade [6]. It is estimated that depression affects 1 in 7 adolescents [6]. Adolescents with depressive symptoms are susceptible to social stigma, discrimination, and cognitive problems. Some studies have shown that the increased use of social media has led to an increase in depressive symptoms [2].

A previous scoping review investigated the association between social media use and depression, examining four factors: quantity of social media use, quality of social networking site use, social aspects of social media use, and disclosure of mental health symptoms on social media [7]. However, this current review extends beyond these factors and includes articles until 2022, while the previous review only included articles until 2020. This review also focuses on the scales used to measure depression and social media use among adolescents and explores new areas of investigation such as gender differences, the impact of social media on sleep quality, and its relationship with depression. It provides an overview of current work and outlines future research questions in the area of social media use and depression among adolescents.

### 1.2. Aim

This scoping review will explore the association between social media and depression among adolescents. The review will consider sources focusing on depression in the specified age group.

## 2. Methods

This scoping review was performed by a team of 6 reviewers using Joanna Briggs Institute (JBI) scoping review method [8]. The scoping review process was carried out using PRISMA-ScR (Preferred Reporting Items for Systematic Reviews and Meta-Analyses Extension for Scoping Reviews) [9]. The review was conducted through five steps: identifying research question, checking relevant studies, study selection, data extraction, and data synthesis.

### 2.1. Search Strategy

#### 2.1.1. Search Source

The following bibliographical databases were searched for the current review: PubMed, Scopus, ProQuest Psychology Database, IEEE Xplore, and Google Scholar. The first ten pages of Google Scholar were scanned as hundreds of citations are usually found there and organized according to relevance. Additionally, other papers were retrieved from the reference lists of the selected papers, and further research pertinent to the evaluation could be identified (backward referencing). Furthermore, forward referencing was conducted to make sure relevant studies were looked at. The search period covered all papers relevant to this study from 2011 until August 2022.

#### 2.1.2. Search Terms

Three criteria were taken into consideration while choosing the search terms for the current review: population (adolescents), intervention (social media, social networks, and media platforms), and results (depression, melancholy, and major depressive disorder). The search terms used to access each electronic database are listed in Supplementary File S1.

### 2.2. Study Eligibility Criteria

**Figure 1:**
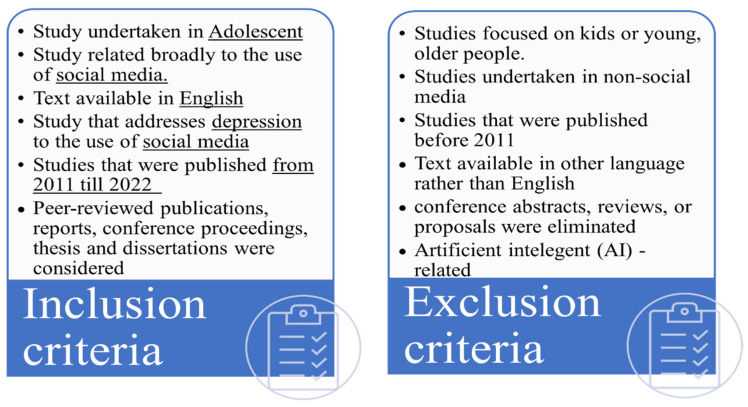
Inclusion and Exclusion Criteria.

Articles met the inclusion criteria if they achieved the main objective, namely studying social media use among adolescents and its possible association with depression and were published between 2011 to 2022. The inclusion and exclusion criteria are listed in Figure 1 below. This review includes peer-reviewed publications, reports, conference proceedings, theses, and dissertations, but it did not include conference abstracts, reviews, or proposals. In studies that included participants of ages more than 19, the determining factor for inclusion was the mean age. Additionally, there were no limitations on the study’s location, gender, research design, stated results, or country of publication.

### 2.3. Study Selection

A two-stage procedure was used to screen every article that was retrieved. First, duplicates were removed. Then, two reviewers read the titles and abstracts of all papers. A review tool, Rayyan [10], was used to speed up the procedure. The Cohen kappa score was used to quantify the interrater reliability between the two reviewers. Reviewer 1 included 139 articles and excluded 456 articles. Reviewer 2 included 143 articles and excluded 451 articles. Reviewer 1 and Reviewer 2 had 6 disagreements. Reviewer 1 wanted to include one paper that reviewer 2 excluded. Reviewer 2 included five articles that reviewer 1 excluded. With this information, we were able to compute the Cohen Kappa score. The Cohen Kappa score was computed to be 0.972. Both reviewers had similar views on most of the papers that needed to be included or excluded. The reviewers solved the issue of disagreement by talking to each other and arriving at a consensus. In the end, the two reviewers agreed to include 138 articles and exclude 457 articles. The inclusion of possibly pertinent items was then assessed by reading the full text of the primarily included papers.

### 2.4. Data Extraction

To identify and analyze results, the reviewers considered 15 categories of data to be extracted from the included papers. The reviewers built the data extraction sheet to manage the obtained information. The categories included author names, country where the study was conducted, publication year, study objective, population size, gender, age range and mean age, data scales used, and published findings. Six impartial reviewers examined the characteristics of the study based on the predetermined classification. Excel was utilized for both synthesis and analysis.

Out of the 43 included papers, reviewers were able to extract data and fill 15 categories which resulted in 645 points of extraction (43 × 15). Reviewers agreed on 610 of the extracted data with no conflict. For the remaining 35 extracted data, reviewers set up a meeting to discuss the outcomes, and were able to consensually agree on the results. Accuracy score for data extraction was 94.6%. 

### 2.5. Data Synthesis

The gathered data were analyzed and presented using narrative synthesis. The included studies and results finding that were addressed in the literatures were compiled in a table in Supplementary File S1.

## 3. Results

### 3.1. Characteristics of the Studies Included

**Figure 2:**
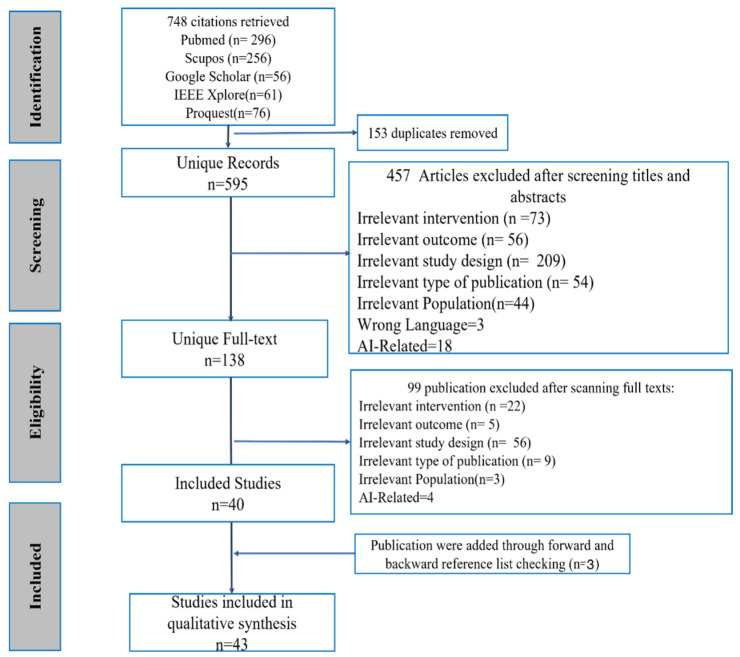
PRISMA Flowchart.

In this scoping review, 748 articles were obtained from five databases (Scopus = 256 articles, PubMed = 296 articles, IEEE Xplore = 61 articles, ProQuest Psychology Database = 76 articles, and Google Scholar = 56 articles,) as shown in Figure 2. Initially, 153 duplicates were removed which resulted in 595 unique articles. The studies were retrieved from various sources, which increased the chance of duplicates. Rayyan was used to screen the articles and remove duplicates in the process. All the selected articles were published between 2012 and 2022. They were written in English and focused on social media use by adolescents and depression. In the first phase of study selection, 457 articles were removed based on the exclusion criteria (irrelevant intervention = 73, irrelevant study = 209, irrelevant outcome = 56, irrelevant population = 44, review papers = 54, AI-related = 18 articles, and non-English articles = 3). In the second phase of the study selection, full texts of the remaining 138 articles were reviewed. Finally, 40 articles were included. Two additional studies were added through forward referencing and one article was added by backward reference checking. In total, 43 articles were selected.

### 3.2. Setting and Research Phase

We included 43 articles for this scoping review. These articles were published in 18 different countries; Romania (1, 2.33%) [11], Australia (3, 6.98%) [12,13,14], Belgium (1, 2.33%) [15], Canada (2, 4.66%) [16,17], China (5, 12.96%) [18,19,20,21,22], Finland (1, 2.33%) [23], India (1, 2.33%) [24], Iran (1, 2.33%) [25], Nigeria (2, 4.66%) [26,27], Norway (3, 6.98%) [28,29,30], Serbia (1, 2.33%) [31], Spain (1, 2.33%) [32], Taiwan (1, 2.33%) [33], Thailand (1, 2.33%) [34], Tunisia (1, 2.33%) [35], Turkey (3, 6.98%) [36,37,38], United Kingdom (4, 9.30%) [39,40,41,42], and United States (11, 25.56%) [43,44,45,46,47,48,49,50,51,52,53]. 

**Figure 3:**
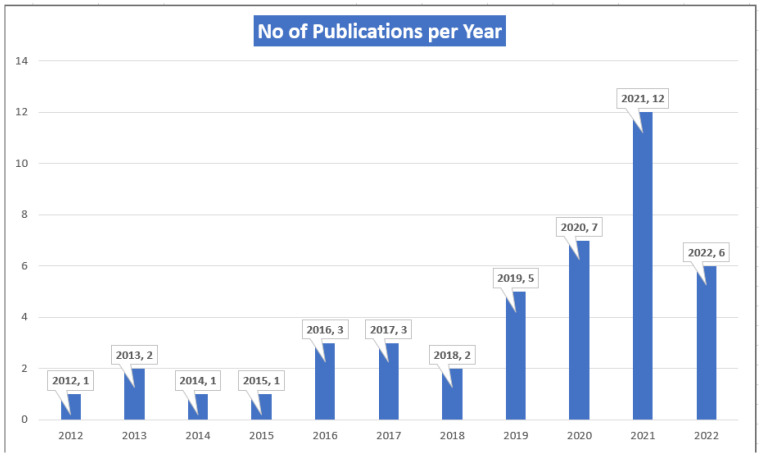
Publication by Year.

The highest number of articles was from the United States as shown in Table 1. Most articles were published in 2021 (12, 27.27%) as shown in Figure 3. We identified four social media platforms that were mentioned in the articles. These included Facebook (4, 9.30%) [35,41,45,53] Instagram (1, 2.33%) [15], multi-platform (37, 86.05%) and Qzone (1, 2.33%) [22].

**Figure 4:**
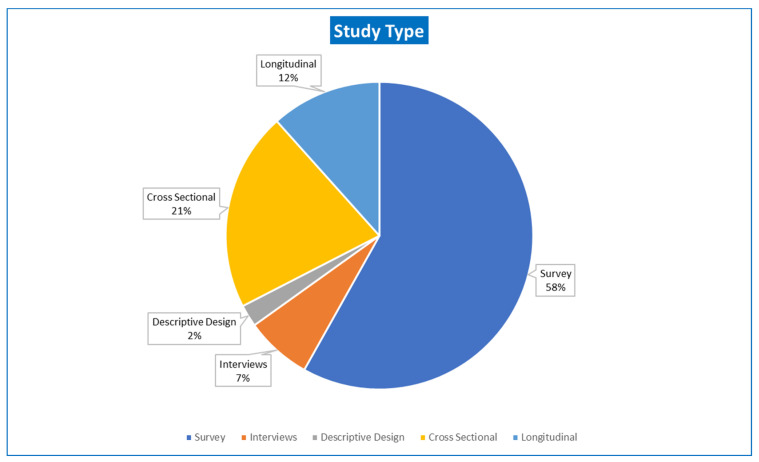
Type of Study.

The articles included various study design types as shown in Figure 4. Around 58% of the included studies involved surveys, while 21% were cross-sectional in nature. Longitudinal studies represented 11% of the studies included, 7% relied on interviews, and 2% were descriptive design studies.

### 3.3. Findings

**Figure 5:**
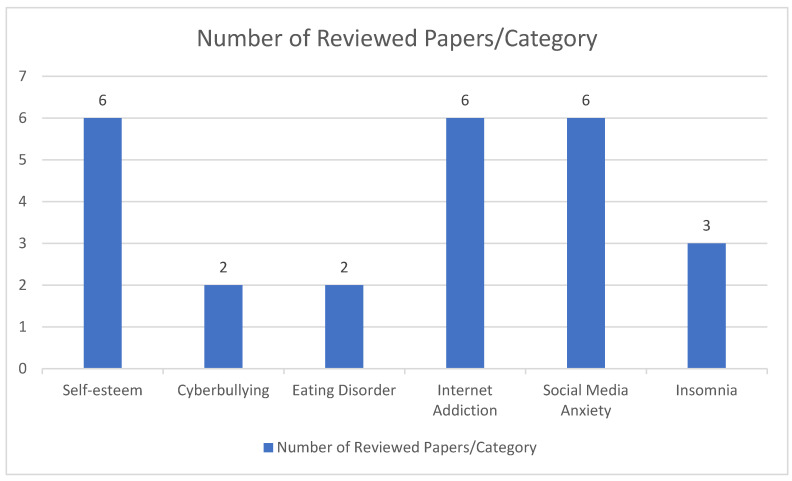
Number of reviewed papers per area.

Some papers concluded an association between social media use and depressive symptoms, in addition to other symptoms such as anxiety, insomnia, lack of self-esteem, social and appearance anxiety, reassurance seeking, and even internet addiction [3,13,14,15,16,18,19,20,21,22,23,24,26,28,29,32,35,36,37,38,39,40,41,43,46,47,50,51,52]. These papers reported that the number of hours spent on social media is associated with an increase in depressive symptoms and other mental health problems among adolescents. Additional factors were taken into consideration such as gender, demographics, cyberbullying, eating disorders, and other addiction problems. However, four of the reviewed articles showed no to minimal or moderate association between social media use and depression in adolescents [27,30,31,53] Below is Figure 5 which shows the number of reviewed papers associated with depression and self-esteem [14,22,25,39,40,47], cyberbullying [34,51], eating disorders [11,12], internet addiction [18,24,33,35,36,38], social media anxiety [2,11,13,39,46,52], and insomnia [25,39,51]. The percentage of the male population in the samples was less than that of the female population in 23 papers [13,14,15,16,20,21,23,27,30,31,32,35,37,38,41,43,46,47,48,49,51,52]. In other papers [11,12,19,25,40,50], the sample of participants was almost equal in number between males and females. The size of population samples in all the reviewed papers ranged between 18 and 74,472. 

Eight articles reported that females who use social media showed higher depression symptoms than males who did [16,18,28,29,40,43,50,52]. Maheux et al. [43] explained in their paper that females have reported a higher overall score on the Appearance-Related Social Media Consciousness scale (ASMC), which explains why females might be more affected by social media use which, therefore, increases their depressive symptoms. It was reported that females spent 7.5 + 4.4 h on average using social media a day. However, males used social media for longer hours, yet they did not show significant depressive symptoms compared to females [43].

Demographic characteristics, family environment, and psychosocial factors, as Zhang et al. [18] showed, were associated with internet gaming addiction, social media addiction, and smartphone addiction. In addition, negative psychological factors such as anxiety and depression play a vital role in different behavioral addictions. According to this study, males have a higher tendency to gaming addiction than females, and internet addiction among adolescents is affected by family environment and demographic factors.

S. Charoenwanit [34] revealed in his paper that 39% of the interviewees, which is more than 1/3 of the sample size, were bullied on social media. Additionally, cyberbullying was associated with academic achievement, general health, and depression among adolescents with a statistical significance of 0.01 for *p*-value. T.D. Ray [47] reported that adolescents experiencing social comparison and cyberbullying during a developmental stage in their life resulted in depression, a lack of self-esteem, and a significant impact on their emotional wellbeing. Shafi et al. [49] highlighted in their paper that social media usage increase to be considered a consequence of depression, and potentially increases the cyberbullying score as well. Many assume that increased social media usage causes depression, but such papers suggest that it is a two-way relationship. Additionally, Ghergu et al. [11] showed that social media use might increase the chances of developing unhealthy eating attitudes, yet it can also play a protective role for those who already developed eating disorders [11].

Pirdehghan et al. [25] showed in their paper that sleep quality had a significant negative correlation with social media use statistically (*p*-value = 0.02), and that males use social media more than females. Thus, males sleeping quality would be lower. Previously in some papers [16,18,28,29,40,43,50,52], it was shown that social media use affected females as they reported higher depressive symptoms; however, Pirdehghan et al. [25] showed that gender does not play a significant role and that the more social media use, the more depressive symptoms are expressed regardless of the gender.

The included papers used different scales and metrics to measure different aspects of mental health that were directly or indirectly associated with depression, anxiety, or both. Only a few papers used similar scales and metrics to measure mental health values. For social media use, there were two main factors to be measured: the number of hours spent on social media, and how social media was used by adolescents. Most papers used questionnaires or self-assessment tools in addition to some pre-identified.

Some papers opted for self-reporting of depression symptoms, anxiety, or other mental health problems through surveys and questioners, in addition to reporting the number of hours spent on social media [12,15,29,30,35,38,44,45,47].

The included papers used different scales to measure depression levels. Only three depression measuring scales appeared to be used in multiple papers. The Hospital Anxiety and Depression Scale (HADS) was used in two papers to assess the level of depression in adolescents [25,27]. M. Culpepper [46], Wang et al. [20], and M. Kwon et al. [51] used the Centre for Epidemiological Studies-Depression (CES-D) scale to test depression levels in adolescents. Furthermore, two studies conducted by Li et al. [21] and G. Niu et al. [22] used an altered version of the Epidemiological Studies-Depression scale to test adolescents for depression. Moreover, the Children’s Depression Inventory scale was used to measure depression among adolescents in the studies conducted by K. Kırcaburun [36] and S. R. Liu et al. [50].

Other scales were used to measure factors that could be associated with depression such as self-esteem, loneliness, sleep quality, and anxiety. Rosenberg Self-esteem Scale is one of the scales that were used by multiple studies to measure self-esteem [17,20,36,39,42,49], in addition to the shortened version of the scale used by D. A. Barthorpe et al. [40] and the Chinese version of the scale that was used by G. Niu et al. [22]. Two studies by S. YAŞAR CAN et al. [37] and S. R. Liu et al. [50] used UCLA Loneliness Scale to measure how disconnected adolescents were feeling and if it was associated with social media user or not. Pirdehghan et al. [25], F. F. Ibimiluyi [27], and M. Kwon et al. [51] included studies about sleep quality and social media use and that could be a possible reason for depression among adolescents. The scale used for the assessment of sleep quality was Pittsburgh Sleep Questionnaire Index (PSQI) [25,27,51]. The Generalised Anxiety Disorder Assessment scale (GAD-7) is a scale used to measure anxiety in the studies by M. Culpepper [46] and W. Zhang et al. [18]. Two studies by Shafi et al. [14,49] measured Salivary cortisol levels to measure anxiety and if it could possibly be associated with social media use.

For measuring social media addiction, Bergen Social Media Addiction Scale (BS-MAS) was used in two studies by W. Zhang et al. [18] and R. M. A. Shafi et al. [49]. Social Function use Intensity (SFUI) scale and Entertainment Function use Intensity (EFUI) scale were two scales that occurred to be used in two papers by A. Ghergut et al. [11] and J.-B. Li et al. [19] to measure social media use and entertainment intensity. Finally, Facebook Intensity Scale (FBI) was used to measure Facebook usage and emotional connectivity to the website alongside other aspects. The FBI scale was used in two papers by T. Hawes et al. [13] and G. Niu et al. [22].

All other scales used to measure social media use, depression, and factors that are possibly associated with depression occurred once as displayed in Table 2. The table concludes that there were many depression evaluation scales used, but only a few scales were used by a multiple of the studies included. Self-esteem, loneliness, sleep quality, and anxiety were factors that appeared to be a concern in multiple studies.

## 4. Discussion

This scoping review aimed to provide an insight on increasing social media use and depression, and to see if these two variables affect each other. Depression was taken into consideration as it is evident how much its rate is increasing. According to K. Kircaburun [36], depression is one of the major health problems in modern society. In 2021, the World Health Organization (WHO) conducted research that revealed that depression is affecting around 350 million worldwide [4]. It is recognized that technology is becoming more of a need than a want day by day, and that it has become the source of income for different influencers and content creators. However, the impact of this needs to be considered and managed as well. It is important to understand that there can be direct impact and indirect impact of social media usage on depression. When there is an indirect impact, it can be due to factors such as decreased physical activity because of spending many hours on social media, emotional eating due to self-esteem and body image issues resulted from social media content, lack of sleep because of prioritizing using social media over sleep quality, internet addiction, or even cyberbullying. The commonality in the scales used to measure those aspects as shown in the previous section indicated that researchers predicted a possible association between social media use, loneliness, self-esteem, sleep quality, anxiety, and depression.

In this scoping review, 43 articles were reviewed, and around 75% of these papers concluded an association between depression and increased social media use. The articles reviewed had different methodologies for testing this association; some were dependent on analyzing interview and questionnaire responses, while others measured increased cortisol levels by taking saliva samples. Two examples that tested the salivary cortisol level in the papers by Shafi et al. [14,49], which was measured, in addition to *α*-amylase levels, in adolescents after using social media to check if social media use caused anxiety. It was found that salivary cortisol and *α*-amylase levels were significantly higher in adolescents with depression but not in healthy control adolescents. This shows us that people with existing depression may face worse symptoms after using social media. On the contrary, social media use can have a positive effect on those who suffer from eating disorders. Ghergut et al. [11] suggested that social networking use might increase the chances of developing unhealthy eating attitudes in adolescents who are not at risk to develop an eating disorder, but, at the same time, it might play a protective role, instead of a harmful one, for adolescents who already developed such symptoms.

One factor to consider is the average age of the samples. Some papers showed a moderate to low association between social media use and depressive symptoms. [27,31,53] The mean age of the samples in those papers was 18, 18.9, and 15.22, respectively. Those mean ages are higher than the mean ages of samples from other papers that showed a higher association between social media use and depression. This indicates that older adolescents are more aware and resistant to the negative aspects of social media than younger adolescents. Older adolescents seem to deal better with social media’s negative side effects than younger adolescents considering the results shown in the three papers mentioned.

It is important to note that the countries with most studies reviewed where China, five papers [18,19,20,21,22], the United States, eleven papers [43,44,45,46,48,49,50,51,52,53], and Norway, three papers [28,29,30], and Australia, three papers [12,13,14], which are four developed countries. The fact that they are developed countries means that the users have mobiles that are connected to Wi-Fi potentially majority of the time, thus social media use is high as discussed by Poushter et al. [54]. This is where identifying such patterns of social media effect is needed to alleviate any potential negative outcomes.

### 4.1. Strengths and Limitations

This review paper presents a comprehensive examination of the latest research on the association between social media use and depression. The scoping review focuses on peer-reviewed articles from databases such as PubMed, IEEE Xplore, Scopus, Google Scholar, and ProQuest Psychology. The paper aims to gain a deeper understanding of the various factors that contribute to depression in relation to social media use, including gender, sleep quality, and self-esteem. The review also summarizes the scales used in the included articles, highlighting similarities and differences, and providing an overview of the most recent findings in the field. By synthesizing the latest research, this review paper aims to provide a valuable resource for researchers and practitioners in the field of mental health.

This scoping review included five databases which could have limited the number of articles. We focused on articles that were published in English. This could potentially mean that we missed relevant studies in other languages. Moreover, our results show that the articles came from 19 countries. The study missed other populations.

### 4.2. Practical and Research Implications

Practical Implications: In this paper, we looked at the possible association between social media use and depression. As such, this review can potentially aid psychologists and mental health experts in gaining insights into the depressive symptoms of adolescent patients. Psychologists and Mental health experts should monitor the relationship between social media use and depressive symptoms as technology continues to rise rapidly. Understanding the link between social media use and depressive symptoms can also lead to better recommendations from mental health experts to aid adolescents. Moreover, this review paper can also help parents assess the effects of social media use on their children.

Research Implications: The review paper looked at several metrics to quantify the amount of depression among social media users. Moreover, the review looked at the factor of gender. Future studies should consider standardizing metrics to quantify depressive symptoms associated with social media use. In our findings, most of the paper used a different scale or metric which made the analysis more tedious. Future studies can also delve deeper into the depressive effect of social media use based on gender. Several studies have demonstrated a possible correlation between social media usage, depression, and gender difference. Some paper demonstrates, for instance, that social media usage affects females more than males. However, we would recommend conducting a systematic review to determine the validity of this relationship.

## 5. Conclusions

This review paper was conducted to explore the link between depression and social media use among adolescents. A total of 43 articles were reviewed, and the highest number of papers came from the US. Furthermore, our analysis looked at several metrics used by researchers to measure depression and other factors that can have an association with it such as self-esteem, eating disorders, sleep, social media anxiety, internet addiction, and cyberbullying. This review suggests that there is an association between social media use and depression among adolescents. It also suggests that social media usage affects females more than males. However, a systematic review needs to be conducted to understand these associations further.

## Figures and Tables

**Table 1:** Characteristics of Studies N = 43.

Country of Publication	Studies (%)	Study ID
Romania	1 (2.23)	[11]
Australia	3 (6.69)	[12,13,14]
Belgium	1 (2.23)	[15]
Canada	2 (4.65)	[16,17]
China	5 (11.62)	[18,19,20,21,22]
India	1 (2.23)	[24]
Iran	1 (2.23)	[25]
Nigeria	2 (4.65)	[26,27]
Norway	3 (6.98)	[28,29,30]
Serbia	1 (2.23)	[31]
Spain	1 (2.23)	[32]
Taiwan	1 (2.23)	[33]
Thailand	1 (2.23)	[34]
Tunisia	1 (2.23)	[35]
Turkey	3 (6.98)	[36,37,38]
United States	11 (25.5)	[43,44,45,46,47,48,49,50,51,52,53]
United Kingdom	3 (6.98)	[39,40,41]
Grand Total	43 (100)	
**Year of Publication**		
2012	1 (2.27)	[31]
2013	2 (4.54)	[24,53]
2014	1 (2.27)	[33]
2015	1 (2.27)	[48]
2016	3 (6.81)	[36,39,41]
2017	2 (4.54)	[15,52]
2018	3 (9.09)	[20,21,22]
2019	5 (11.36)	[17,30,34,42,51]
2020	7 (15.9)	[12,13,23,27,35,40,46]
2021	12 (27.27)	[14,16,25,26,28,29,32,37,38,44,45,47]
2022	6 (13.63)	[11,15,18,19,32,43]
**Social Media Platform**		
Facebook	4 (9.09)	[35,41,45,53]
Instagram	1 (2.27)	[15]
Multi-platform	37 (86.36)	[11,12,13,14,16,17,18,19,20,21,23,24,25,26,27,28,29,30,31,32,33,34,36,37,38,39,40,42,43,44,46,47,48,49,50,51,52]
q-zone	1 (2.27)	[22]

**Table 2:** Scales and Metrics Used.

Scales	Study ID	Social Media Related	Depression/Mental Health Related Factors
Self-assessment—not pre-identified surveys	[12,15,29,30,35,38,44,45,47]	X	X
Eating attitudes test	[11]		X
Revised Child Anxiety and Depression Scale (RCADS-25)	[11]		X
Internet Gaming Disorder Scale- Short Form (IGDS9-SF)	[18]	X	
Smartphone Application- Based Addiction Scale (SABAS)	[18]	X	
Bergen Social Media Addiction Scale (BSMAS)	[18,49]	X	
Strengths and Difficulties Questionnaire-students (SDQ–S)	[18]		X
16-Item Version of the Prodromal Questionnaire (PQ-16)	[18]		X
Multidimensional Peer Victimization Scale (MPVS)	[18]		X
Questionnaire-9 (PHQ-9)	[18]		X
Warwick-Edinburgh Mental Well-being Scale (WEMWBS)	[18]		X
Connor-Davidson Resilience Scale (CD-RISC10)	[18]		X
Appearance-related social media consciousness (ASMC)	[43]	X	X
Depression (Beck)	[25]		X
Social Media Addiction scale	[36]	X	
Pittsburgh Sleep Questionnaire Index (PSQI)	[25,27,51]		X
Children’s Depression Inventory scale	[36,50]		X
Rosenberg Self-esteem Scale	[17,20,36,39,42,49]		X
Chinese version of the ten-item Rosenberg self-esteem scale	[22]		X
Self-esteem (shortened Rosenberg)	[40]		X
UCLA Loneliness Scale	[37,50]		X
Social Media Addiction nScale for Adolescents	[37]	X	
Reynolds Adolescent Depression Scale	[37]		X
Social Anxiety Scale for Adolescents (SAS)	[46]		X
The Generalised Anxiety Disorder Assessment scale (GAD-7)	[18,46]		X
The Centre for Epidemiological Studies-Depression (CES-D) scale	[20,46,51]		X
20-item Chinese version of the Center for Epidemiology Scale for Depression (CES-D)	[21,22]		X
Subscale of the Depression, Anxiety and Stress Scale (DASS-21—Spanish version)	[32]		X
Spanish version of the Wong and Law Emotional Intelligence Scale (WLEIS)	[32]		X
Social Media Addiction Questionnaire (SMAQ)	[32]	X	
Short version of the Perceived Stress Scale (PSS)	[32]		X
Brief Symptom Inventory (BSI)	[16]		X
Social Media and Depression Scale (SMDS)	[27]		X
The Hospital Anxiety and Depression Scale (HADS)	[27,52]		X
Social Media Use Integration Scale	[39]	X	
Social media screen-time as recorded in time use diaries (TUD)	[40]	X	
Back Depression Inventory—second edition (BDI-II-II) scale	[31]		X
The Electronic Interaction Scale for Time (EIS_T)	[48]	X	
Reassurance-Seeking Scale (RSS)	[48]		X
The Motivations for Electronic Interaction Scale (MEIS)	[48]	X	X
The Short Mood and Feelings Questionnaire (SMFQ)	[48]		X
Hopkins Symptom Checklist scale	[28]		X
Salivary cortisol level	[14,49]		X
Quality of Life Scale for Children-Turkish version	[38]		X
Time spent using (Social Networking Services) SNS (internet addiction—AI) and 21 item Depression Anxiety and Stress scale	[24]	X	X
Online Social Networking Activity Intensity Scale (OSNAI) scale	[19]	X	
Social Networking Activity Intensity Scale (SNAI) scale	[11]		
Online social networking addiction (OSNA)	[53]	X	X
Social Function use Intensity (SFUI)	[11,19]	X	X
Entertainment Function use Intensity (EFUI)	[11,19]	X	X
Mini-International Neuropsychiatric Interview for Children and Adolescents (MINI-KID)	[49]		X
Illinois Bully Scale	[49]		X
17-item Clinician Rated Quick Inventory of Depressive symptoms (QIDS-A17-C)	[49]		X
Youth Self-Report Baseline Form	[50]		X
Emotional Connection (EC) meters	[52]		X
Social Networking Intensity scale (SNI)	[52]	X	
Cyber aggression scale	[51]	X	X
Fear of missing out scale (FOMOs)	[52]		X
Mood and Feelings Questionnaire—short version (SMFQ)	[42]		X
Facebook Intensity Scale (FBI) modified	[13,22]	X	X
The Facebook Intrusion Questionnaire	[20]	X	
Ruminative Response Scale (RRS)	[20]		X
The Finnish version of the Depression Scale	[23]		X
Patient Health Questionnaire-9 depression screen (PHQ)	[53]		X
Family Affluence Scale for Adolescents	[17]		X

## Data Availability

The data that support the findings of this study are available from the corresponding author upon reasonable request.

## Supplementary Materials

The following supporting information can be downloaded at: https://www.mdpi.com/article/10.3390/bs13060475/s1, File S1: Search terms and details of included studies.
